# Study on mechanism of temperature-modulated polyphenolic biosynthesis in cigar tobacco leaves

**DOI:** 10.3389/fpls.2025.1693512

**Published:** 2025-10-23

**Authors:** Jiahao Kang, Bo Fu, Xiaoxiao Yan, Menglan Xiao, Weili Yang, Mingqin Zhao

**Affiliations:** ^1^ College of Tobacco Science, Flavors and Fragrance Engineering & Technology Research Center of Henan Province, Henan Agricultural University, Zhengzhou, China; ^2^ Sichuan Provincial Branch of China National Tobacco Corporation, Dazhou, China

**Keywords:** cigar tobacco, long-term, Phenylpropanoid biosynthesis, RNA-seq, temperature stress, WGCNA

## Abstract

Cigars as an economic crop, inappropriate growth temperature can detrimentally affect cigar quality, yet our knowledge about the response of cigar tobacco leaves (CTLs) to such stressors remains limited. We subjected CTLs to prolonged mild low-temperature and high-temperature treatments and systematically assessed oxidative stress markers, antioxidant enzyme activities, phenolic and flavonoid accumulation, and transcriptomic dynamics. Weighted gene co-expression network analysis (WGCNA) and correlation-based approaches were used to identify key genes and regulators.This study revealed that both long-term mild low and high temperatures in CTLs triggered excessive production of reactive oxygen species (ROS) and disrupted antioxidant enzyme systems. Exposure to low temperatures resulted in increased accumulation of phenolic and flavonoids, while high temperatures were associated with either negative or negligible effects on these metabolites. Moreover, low temperatures significantly stimulated phenylpropanoid biosynthesis, in contrast to the negative effects observed at high temperatures. Correlation analysis indicated that *NtMYB12-1*, *NtMYB12-2*, and *NtMYB12-3* likely acted as crucial regulators in the modulation of enzymes involved in phenylpropanoid biosynthesis under variable temperature conditions. Furthermore, weighted co-expression network analysis identified that *NtCHS-6*, *Nt4CL-6*, and *NtPAL-1* worked as central hub genes in phenylpropanoid biosynthesis-related modules. This study provides a deeper understanding of the complex theoretical framework of phenylpropanoid biosynthesis in CTLs when exposed to fluctuating temperatures, offering valuable insights for the cultivation of high-quality cigar products.

## 1 Introduction

Temperature significantly affects plant growth, distribution, productivity, and survival. The growing concern over global climate change draws attention to the detrimental impacts of inappropriate temperatures on crops (Pearce, 2001; Chen et al., 2022). Research into plant responses to temperature stress has mainly focused on understanding biochemical changes, metabolic regulation, and transcriptional modifications.

Both low and high temperatures can trigger the overproduction of reactive oxygen species (ROS), which leads to peroxidation of membrane lipid and subsequent damage to the internal structure of plant cells (Wang et al., 2014; Quan et al., 2023). To counteract these stresses, plants have evolved complex defense mechanisms, including the activation of antioxidant enzymes and other protective components. Enzymes such as superoxide dismutase (SOD), peroxidase (POD), and catalase (CAT) are synthesized to remove excess ROS in plant cells under temperature stress, thereby mitigating lipid peroxidation (Suzuki and Mittler, 2006; Karami-Moalem et al., 2018). Additionally, polyphenols are recognized as key antioxidants in protecting against temperature-induced stress owing to their capability to neutralize ROS (Schulz et al., 2016; Sharma et al., 2019; Reine et al., 2022). The widely studied plant metabolites, including phenolic acids, flavonoids, and lignans, are synthesized through the phenylpropanoid pathway and become activated in unfavorable environmental conditions (Šamec et al., 2021). Their essential roles in alleviating temperature stress have been well-documented; for instance, an increase in flavonoids and anthocyanins has been linked to enhanced freezing tolerance in Arabidopsis under cold stress (Schulz et al., 2016). Furthermore, chlorogenic acid (CGA) has been shown to provide thermotolerance in plants facing both heat and cold stress (Reine et al., 2022).

The CBF-COR signaling pathway is one of the most comprehensively studied mechanisms in response to cold stress. The C-repeat Binding Factor/Dehydration-responsive Element Binding Protein 1 (CBF/DREB1) genes are activated under cold conditions and bind to the promoters of cold-responsive (COR) genes, thereby regulating downstream responses that significantly enhance freezing tolerance (Zhu, 2016). Cold stress can affect membrane fluidity, which may be sensed by plasma membrane-associated proteins, such as calcium channel proteins and COLD1, resulting in a temporary rise in cytoplasmic calcium levels. This calcium influx triggers the activation of calcium-responsive protein kinases (including CPKs, CIPKs, and CRLK1) as well as the MAP kinase (MPK) signaling cascade. The activated MPKs, together with phospholipases, induce the expression of various downstream COR genes (Zhu, 2016). COR proteins play a crucial role in regulating the levels of different osmoprotectants, helping to maintain cell turgor and prevent chilling injury during cold stress (Theocharis et al., 2012).

The expression of heat shock transcription factors (HSF) and heat shock protein (HSP) genes is central in responding to high-temperature stress. Under heat stress, HSPs are produced, functioning as molecular chaperones that prevent protein denaturation and aggregation, thereby increasing plant thermotolerance (Driedonks et al., 2015). HSFs control the expression of HSPs and are key mediators of the plant’s heat stress response. Among these, HSFA has been identified as a major regulator involved in signal perception, transduction, and regulation of heat stress-responsive genes. Additionally, HSFs can activate detoxifying enzymes, such as ascorbate peroxidase (APX) and superoxide dismutase (SOD), to scavenge ROS (Guo et al., 2016; Driedonks et al., 2015). Furthermore, the expression of HSP genes can also be influenced by heat-activated MAPKs via calcium signaling. Various plant hormones are also implicated in response to cold and heat stress, through the regulation of the CBF-COR signaling pathway, ROS balance, or HSP expression (Raza et al., 2023).

Tobacco (*Nicotiana tabacum* L.) is susceptible to temperature fluctuations. The growth, development, and polyphenol metabolism of tobacco are regulated by temperature. High temperatures induce ROS accumulation, leading to accelerated flowering, senescence, and a decrease in polyphenol content. Conversely, these processes are inhibited under low-temperature conditions (Yang et al., 2018). Additionally, when tobacco fields experience low temperatures, the leaves undergo discoloration, developing large brown areas and exhibiting poorer curing characteristics. Further studies reveal that this is due to the disruption of the leaf’s antioxidant enzyme system by the cold stress, which ultimately leads to significant declines in yield, quality, and the content of key chemical components (Li et al., 2021). Despite some research on short-term temperature stress, the effects of prolonged exposure on tobacco, especially at the transcriptional level, remain underexplored. Moreover, cigar tobacco, distinguished by their exceptional taste and quality, has gained popularity in the domestic tobacco market. However, the responses and underlying mechanisms of cigar tobacco to such conditions are not well-documented. This research focused on examining the physiological and metabolic responses of cigar tobacco leaves (CTLs) under prolonged exposure to both low and high temperatures. Furthermore, transcriptomic profiling and the development of a stress-responsive regulatory network have provided deeper insights into the molecular mechanisms underlying CTL responses to temperature stress. This study will lay the groundwork for the breeding of cigar tobacco varieties that are both resilient and of superior quality.

## 2 Materials and methods

### 2.1 Plant materials and growth conditions

The Chinese Sichuan cigar variety, Dexue No. 3, served as the plant material for this study. Seeds were sown in trays and grown in climate room equipped with natural lights for illumination. After 40 days of sowing, cigar seedlings were transplanted into pots. When the first true leaf was developed, identical seedlings were selected for stress treatments. Each 15 pots were transferred into climate chambers at 16°C (L, low temperature),24°C (M, normal temperature), and 32°C (H, high temperature) separately.

All other environmental conditions in the climate rooms remained consistent, with natural light provided or supplemented by indoor high-pressure sodium lamps when natural light was insufficient. These conditions included a 14-hour light/10-hour dark photoperiod, a light intensity of 300 μmol·m^-2^·s^-1^ measured on the 12th leaf from the bottom of the plants, 60% relative humidity, and a CO_2_ concentration of 400 mmol·mol^-1^. Samples were collected at distinct growth stages, namely the rapid growth (RG) stage (40 days of stress duration) and the physiological maturity (PM) stage (60 days of stress duration).

### 2.2 Measurement of morphological, physiological, and biochemical parameters

The 12th leaves from the bottom up of cigar tobacco plants were collected for morphological and physiological measurements. Leaf Area Index (LAI) was determined by the following formula: LAI = (Total leaf area)/(Soil area). The net photosynthetic rate (Pn) of the CTLs was measured at 9:00 -10:00 am using the portable photosynthesis measurement instrument GFS-3000(WALZ GmbH, Germany). The H_2_O_2_, O_2_^·−^, MDA content, as well as SOD, POD, CAT, and Rubisco were quantified using specific assay kits from Solarbio Company. For each treatment, we used 15 seedlings, with three replicates per treatment and five seedlings per replicate.

### 2.3 Measurement of secondary metabolites in cigar leaves

The total phenolic contents (TPCs) were assessed using the Folin–Ciocalteu colorimetric method as described by Lee et al. (2012). For the quantification of total flavonoid compounds (TFCs), a colorimetric approach was applied according to the protocols of Dewanto et al. (2002). The contents of chlorogenic acid, rutin, and scopoletin were analyzed using reversed-phase high-performance liquid chromatography (HPLC), following the method established by Scarano et al. (2020).

### 2.4 RNA-seq analysis

The 12th leaves from the base of cigar tobacco plants cultivated at three different temperatures-16°C (low, L), 24°C (medium, M), and 32°C (high, H) were harvested for RNA extraction. Three biological replicates were utilized, each consisting of samples from five individual plants. RNA was extracted using the mirVana miRNA Isolation Kit (Ambion) following the manufacturer’s protocol. The RNA’s quality and purity were assessed using a Thermo Scientific Nano Drop 1000 Spectrophotometer, ensuring that the A260/A280 and A260/A230 absorbance ratios were approximately 2.0. Additionally, the RNA sample integrity was evaluated with an Agilent 2100 Bioanalyzer (Agilent Technologies, Santa Clara, CA, USA), with all samples showing an integrity number above 7.0, making them suitable for RNA sequencing.

Transcriptome sequencing was carried out by OE Biotech Co., Ltd. (Shanghai, China). Library construction was performed using the TruSeq Stranded mRNA LT Sample Prep Kit (Illumina, San Diego, CA, USA) according to the kit’s instructions. Sequencing was then performed on the Illumina platform, generating 125 bp/150 bp paired-end reads. Gene expression levels, expressed in fragments per kilobase of transcript per million mapped reads (FPKM), were calculated using Cufflinks software. Differentially expressed genes (DEGs) were identified based on Padj<0.05 and |log2foldchange|>1, using the DESeq R package. These DEGs were then annotated to Gene Ontology (GO) categories, including biological processes, molecular functions, and cellular components, and subjected to KEGG pathway enrichment analysis using the ClusterProfiler R package.

### 2.5 Weighted gene co-expression network analysis

Through WGCNA analysis, genes with similar expression patterns were clustered to identify co-expressed gene modules. These modules were then correlated with the polyphenol content and enzyme activity in tobacco leaves under different temperatures to identify the core genes associated with them.Gene co-expression networks were constructed using the WGCNA package (Version 4.0.2) within the R environment. Genes with low expression levels (FPKM < 1) were filtered out before network generation. An adjacency matrix was created using a soft-thresholding power (β) of 14. To identify co-expression patterns, the Topological Overlap Matrix (TOM) was computed, and hierarchical clustering was applied to the TOM using the dynamic tree cut algorithm to group genes into distinct modules. Pearson correlations were subsequently calculated between each module and the genes or metabolites involved in phenylpropanoid biosynthesis. Network visualization for the modules of interest was conducted using Cytoscape software (version 3.6.1) with a weight parameter cut-off value set at 0.2 (Langfelder and Horvath, 2008). Hub genes were defined based on their module eigengene connectivity (KME), with those showing an absolute KME value of 0.7 or higher considered as hub genes.

### 2.6 Real-time qPCR

The RNA extraction and quality assessment were performed according to the procedures outlined in section 2.5. After removing genomic DNA contaminants, the first-strand cDNA was synthesized through reverse transcription. Quantitative real-time PCR (qRT-PCR) was carried out using an ABI StepOne Plus Real-Time PCR System with the Green Supermix kit (Shanghai, China). Primers were designed online using Primer 5 software, as indicated in **Supplementary Table S1**, with UBI employed as the reference gene. The relative expression levels of the target genes were measured from three independent biological replicates and calculated using the 2^^-ΔΔCT^ formula.

### 2.7 Statistical analysis

Significant differences were determined by a one-way ANOVA followed by Tukey’s *post-hoc* test (P < 0.05). All statistical analyses were conducted using SPSS v20 (IBM, NY, USA).

## 3 Results

### 3.1 Physiological and biochemical response to low/high temperature in CTLs

**Figure 1:**
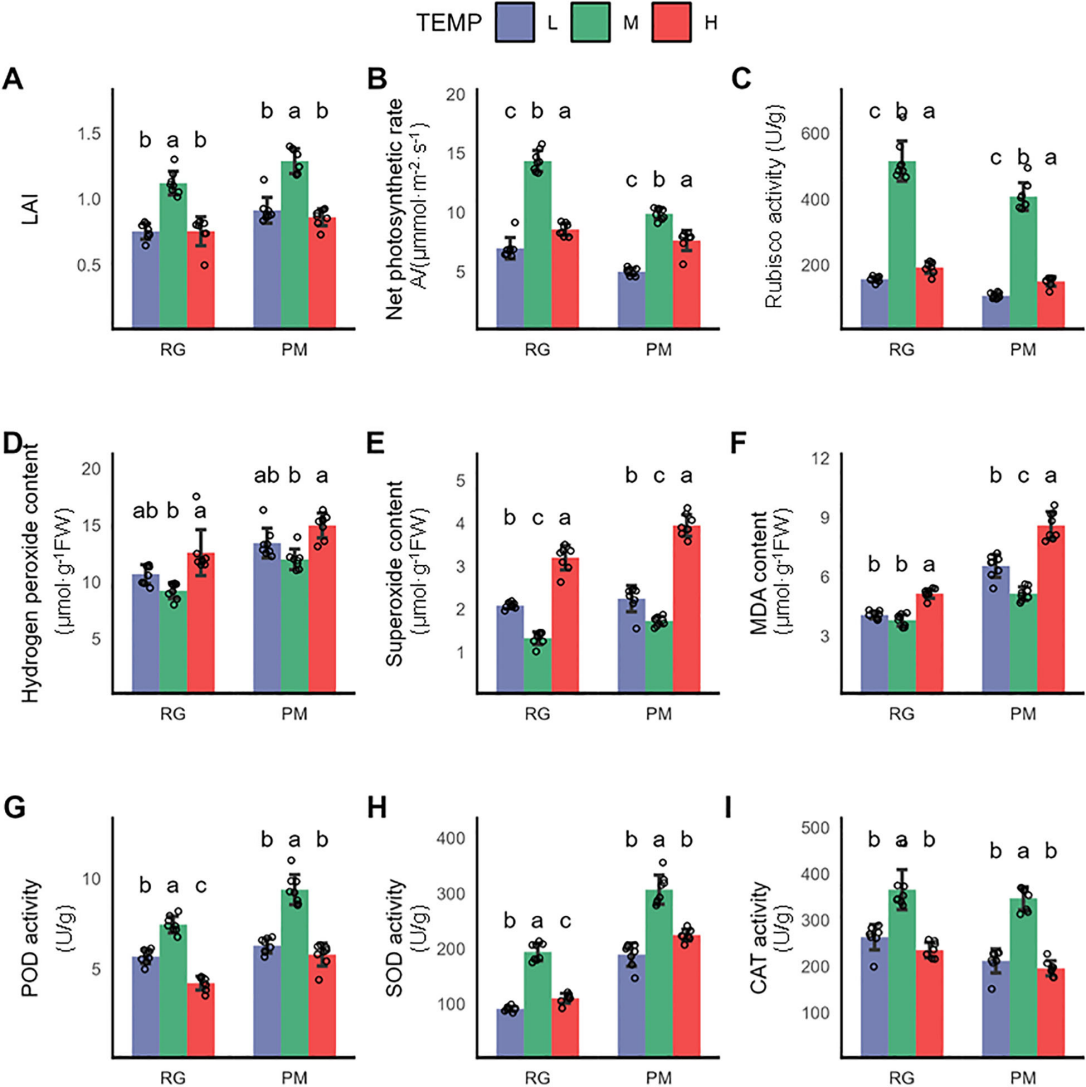
Physiological changes in CTLs exposed to low and high temperature terminated at RG stage and PM stage. **(A)** Leaf area indea (LAI), **(B)** Net photosynthetic rate (Pn), **(C)** Rubisco activity, **(D)** Hydrogen peroxide content, **(E)** Superoxide anion content, **(F)** MDA content, **(G)** POD activity, **(H)** SOD activity, **(I)** CAT activity. Values indicate the mean ± SD (n=3), each sample was represented by empty circle. Statistical analysis of differences was performed using one-way ANOVA, with subsequent Tukey *post-hoc* testing. Significant differences are denoted by distinct letters, indicating a p-value of less than 0.05.

To investigate the effects of low (16°C, referred to as “L”) and high temperature (32°C, referred to as “H”) on the growth of CTLs, we compared the physiological and metabolic changes in CTLs with those observed under control conditions (24°C, referred to as “M”).Exposure to both long-term low and high temperatures significantly inhibited plant growth, as evidenced by a marked reduction in the Leaf Area Index (LAI) compared to the control (**Figure 1A**). This reduction directly indicates impaired leaf expansion, which can compromise light interception and overall biomass accumulation. More critically, we observed a significant decline in the net photosynthetic rate (Pn) and the activity of Rubisco(Ribulose-1,5-bisphosphate carboxylase/oxygenase), the key enzyme in carbon fixation (**Figures 1B, C**). These findings suggest that temperature stress severely disrupts the photosynthetic apparatus, likely limiting the energy and carbon skeletons required for growth and the synthesis of defense compounds, such as polyphenols. Plants under abiotic stress often experience an imbalance in reactive oxygen species (ROS) metabolism. We therefore quantified the levels of hydrogen peroxide (H_2_O_2_) and the superoxide anion (O_2_^•−^), along with malondialdehyde (MDA), a reliable marker for oxidative damage to membrane lipids,leading to oxidative stress marked by increased malondialdehyde (MDA) levels- a byproduct of lipid peroxidation (Suzuki and Mittler, 2006). Our findings showed stable hydrogen peroxide (H_2_O_2_) levels under low temperature, but a significant rise in superoxide anion (O_2_^•−^) levels, with MDA levels increasing significantly at the PM stage (**Figures 1D–F**). In contrast, high-temperature stress elicited a more pronounced accumulation of H_2_O_2_, O_2_^•−^, and MDA, suggesting more severe oxidative stress (**Figures 1D–F**). This was paralleled by a significant reduction in the activities of antioxidant enzymes (**Figures 1G–I**), including POD, SOD, and CAT, indicating that the inherent antioxidant system in plants may be inadequate for effectively counteracting induced oxidative stress.

### 3.2 The response of polyphenolic metabolites to low/high temperature in CTLs

**Figure 2:**
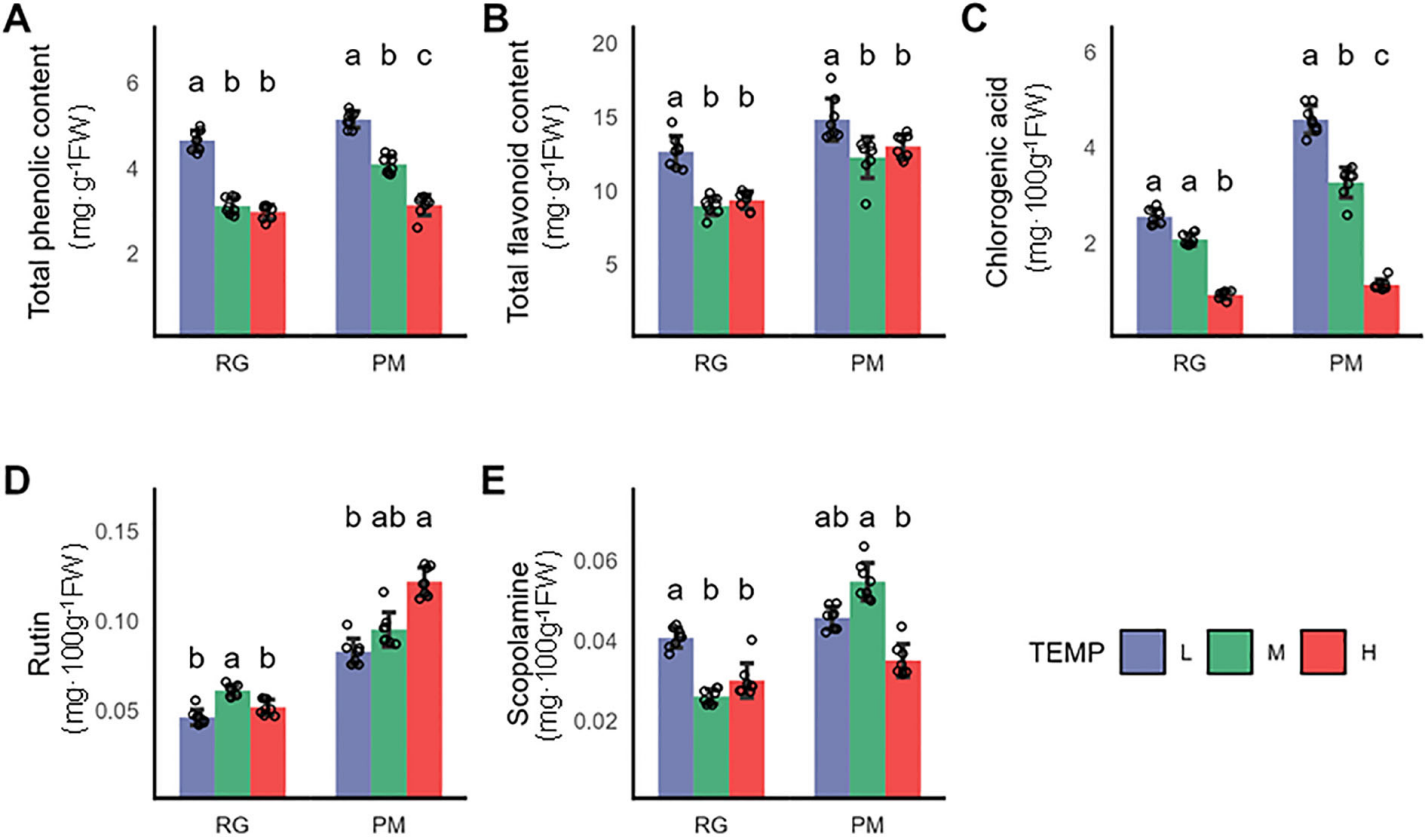
Impact of varying growth temperatures on polyphenolic metabolite levels in CTLs. **(A)** Total phenolic content, **(B)** Total flavonoid content, **(C)** Chlorogenic acid levels, **(D)** Rutin concentration, **(E)** Scopoletin levels. Values represent the mean ± SD (n=3). Statistical differences were assessed using one-way ANOVA followed by Tukey’s *post-hoc* test. Distinct letters denote statistically significant differences (P < 0.05).

Polyphenolic compounds significantly contribute to plant resilience against environmental stress while also impact the essential quality characteristics of tobacco products, including color, flavor, and physiological integrity (Zou et al., 2021). This study investigated the changes of polyphenolic compounds in CTLs subjected to both low and high-temperature treatments. Chlorogenic acid emerged as the primary polyphenolic compound in CTLs, followed by rutin and scopoletin (Sharma et al., 2019). Remarkably, the chlorogenic acid level in CTLs subjected to low temperature until the PM stage was 1.4-fold higher compared to those grown under normal temperatures (**Figure 2C**). While a minor reduction in rutin content was noted at the RG stage, it became statistically insignificant by the PM stage (**Figure 2D**). Rutin content decreased significantly after the stress terminated at the RG stage. Additionally, scopoletin levels in CTLs rose by 36% post-stress at the RG stage but this increase reverted with prolonged stress until the PM stage (**Figure 2E**). A significant improvement in the overall phenolic and flavonoid content was observed in CTLs at both the RG and PM growth stages following low-temperature exposure. Conversely, under high temperature, only a slight reduction in phenolic content was apparent at the PM stage, with flavonoid levels remaining stable across both stages. Chlorogenic acidshowed reductions of 56.2% and 65.7% when stressed until the RG and PM stages, respectively, due to its sensitivity towards temperature fluctuations (**Figure 2C**). The response of rutin content to high temperature followed a pattern similar to its response to cold stress (**Figure 2D**). As for scopoletin, a significant decrease of 24% was exclusively observed at the PM stage under high temperature conditions (**Figure 2E**).

### 3.3 Analysis of the aroma components in CTLs after exposure to low and high temperature

**Figure 3:**
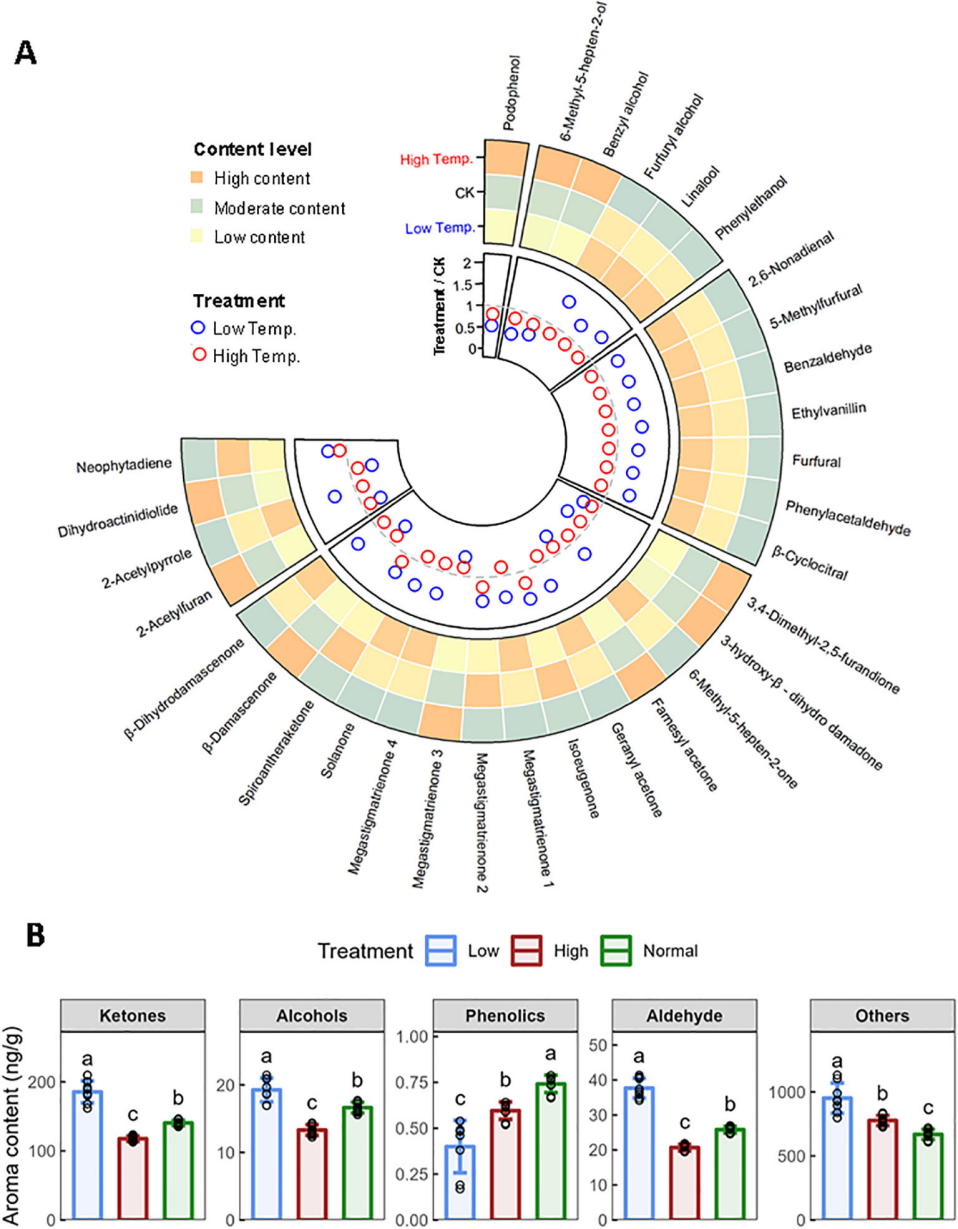
Effects of low temperature and high temperature on aroma components in flue-cured CTLs. **(A)** Comparison of contents of neutral aroma components in different temperatures. **(B)** Changes of various types of aroma components. Values indicate the mean ± SD (n=3). Differences were analyzed by one-way ANOVA followed by Tukey *post-hoc* test. Different letters indicate significant difference (P<0.05).

This study investigated the effects of temperature on the profile of aroma compounds in flue-cured CTLs, identifying 31 aroma components, including alcohols, aldehydes, ketones, and phenolics and others. We observed that low temperatures enhanced 68% of these components, whereas high temperatures decreased 87% of them (**Figures 3A, B**). The total contents of alcohols, ketones, and aldehydes were elevated significantly by low temperature but decreased by high temperature. Ketones represented the most substantial category, with nine types markedly enhanced, as opposed to only three at elevated temperatures (**Figure 3A**). Solanone, the predominant ketone and second most significant aroma compound, saw an increase at low temperatures, probably contributing to an enriched and complex flavor profile. In contrast, podophenol, the only detected phenolic volatile, exhibited declines under both temperature stresses. Moreover, neophytadiene, as the most abundant volatile in CTLs, increased by 43.0% at low temperatures and by 16.6% at higher temperatures, potentially mitigating smoke irritation and fostering a smoother experience. The results indicated that low temperatures improved essential aromatic components, potentially elevating the aromatic quality of cigars, contrary to the effects of high temperatures.

### 3.4 Transcriptome profiles of CTLs subjected to low and high temperature

**Figure 4:**
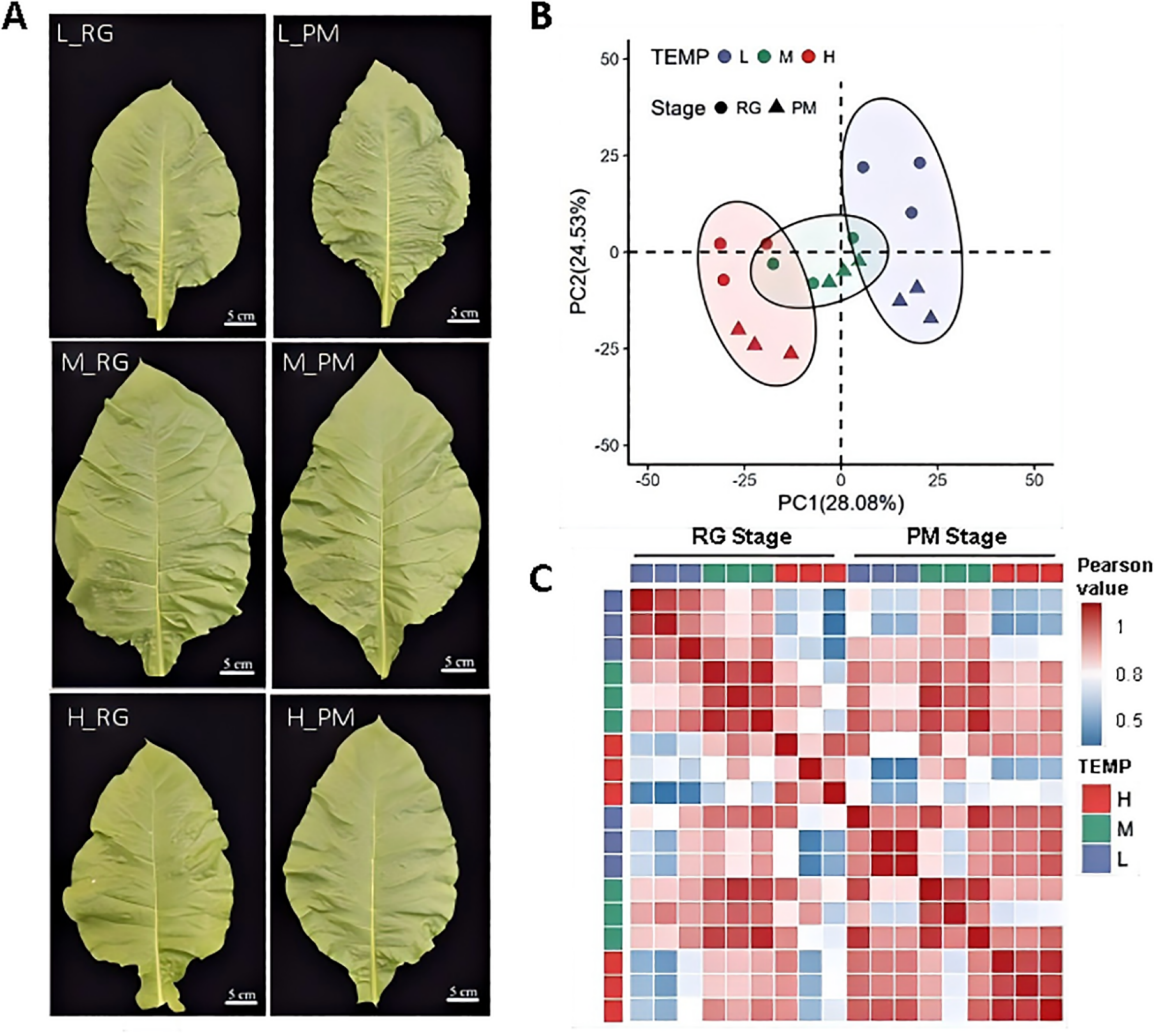
Transcriptomic profiles of leaves exposed to low temperature (L), normal temperature (M) and high temperature (H). **(A)** Morphological observation of CTLs for RNA-Seq analysis. **(B)** Characterization of transcriptome data of CTLs treated with three different temperature treatments till RG stage and PM stage by PCA. **(C)** Pearson correlation matrix of gene expression across all the samples.

To elucidate the molecular mechanisms underlying CTLs’ responses to various temperature conditions, leaves were collected from cigar tobacco seedlings exposed to 16°C(L), 24°C(M), and 32°C (H) until the RG and PM stages for RNA-Seq analysis (**Figure 4A**). After removing low-quality reads, poly-N sequences, and adaptor sequences, a total of 109.34 GB of clean data was obtained (**Supplementary Table S2**). The filtered samples comprised an average of 6.07GB of high-quality data with an average Q30 base percentage of 94.01% and an average GC content of 43.36% (**Supplementary Table S2**). Notably, between 97.56% to 98.0%,the clean reads aligned successfully to the Nicotiana tabacum reference genome, with a uniquely mapping rate ranging from 86.14% to 87.29%. (**Supplementary Table S3**).

Principal component analysis (PCA) of genes with FPKM >0.5 demonstrated distinct separations based on temperature treatments, with PC1 and PC2 accounting for 28.08% and 24.53% of the variance, respectively (**Figure 4B**). Interestingly, while PC1 indicated no significant stage differences, PC2 revealed significant variations between RG and PM stages under both temperature treatments. A Pearson distance matrix highlighted strong correlations among replicates for each temperature treatment, validating the high quality of the transcriptomic data (**Figure 4C**). The RG and PM stages showed weaker correlations under high and low temperatures compared to normal temperature conditions, highlighting significant transcriptional responses to varying durations of temperature stress. These findings are consistent with previously observed physiological variations (**Figures 1**, **2**). At the RG stage, a stronger correlation was observed between M and L treatments than between M and H, indicating that high temperatures might lead to more substantial transcriptional adjustments at this developmental stage. By the PM stage, both L and H showed similar correlations with M, underscoring their equivalent impact on transcriptional levels. Furthermore, the high correlation between L and H at the PM stage suggested that both temperature stresses elicited comparable transcriptional responses at this point in development.

### 3.5 Differentially expressed genes in response to low/high temperature

**Figure 5:**
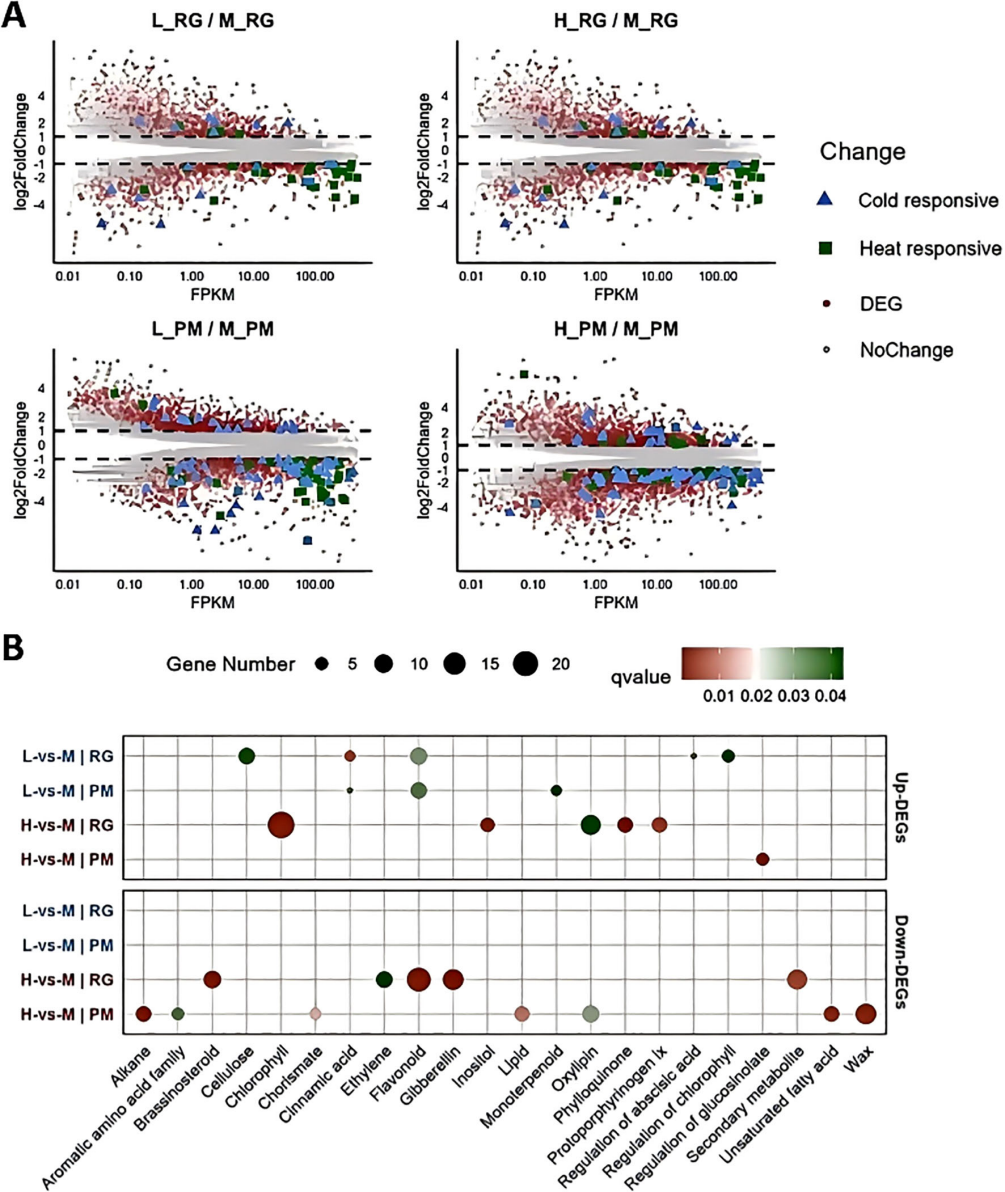
Identification and functional analysis of differentially expressed genes (DEGs) **(A)** MA plot showing differential expression levels of genes across various treatments. The x-axis represents the expression intensity (FPKM), while the y-axis (log2 FC) indicates the fold change in gene expression levels. Each dot represents an individual gene: red dots denote DEGs, grey dots indicate non-differentially expressed genes, blue triangles signify cold-responsive genes, and green dots represent heat-responsive genes. **(B)** GO functional analysis displaying the most representative GO terms for both up-regulated and down-regulated DEGs across all treatments.

To decipher the molecular basis of responses in CTLs to low and high temperatures, differentially expressed genes (DEGs) were identified (**Supplementary Table S4**). At the RG and PM stages, we identified 4,346 and 2,788 DEGs in response to low temperature, as well as 2,443 and 1,599 DEGs in response to high temperature (**Figure 5A**, **Supplementary Table S4**). We further explored the transcriptional dynamics of previously annotated cold-responsive and heat-responsive genes, and observed a predominant trend towards downregulation in both conditions. Notably, the progression to the PM stage induced a broader activation of temperature-responsive DEGs (**Figure 5A**, **Supplementary Table S5**).

Gene Ontology (GO) enrichment analysis further illuminated the biological processes implicated in these responses (**Supplementary Table S6**, **Supplementary Figure S1**). Under low temperatures, upregulated DEGs at the RG stage were significantly associated with pathways, such as cellulose biosynthesis, flavonoid production, chlorophyll regulation, and cinnamic acid metabolism, shifting towards flavonoid and monoterpenoid processes by the PM stage (**Figure 5B**). However, downregulated DEGs at both stages were mainly enriched in the carbohydrate binding term (**Supplementary Figure S1**). In contrast, under high temperature, up-regulated DEGs at the RG stage were enriched in functions related to chlorophyll biosynthesis and oxylipin metabolism (**Figure 5B**). As exposure extended to the PM stage, the up-regulated DEGs were primarily associated with the regulation of glucosinolate metabolism. Downregulated DEGs under high temperature were significantly enriched in flavonoid biosynthesis, secondary metabolites, gibberellin and brassinosteroid signaling, and ethylene regulation at the RG stage, but shifted focus to wax biosynthesis, oxylipin metabolism, alkane production, and lipid metabolism by the PM stage (**Figure 5B**).

### 3.6 Regulation of phenylpropanoid biosynthetic pathway under low and high temperature

**Figure 6:**
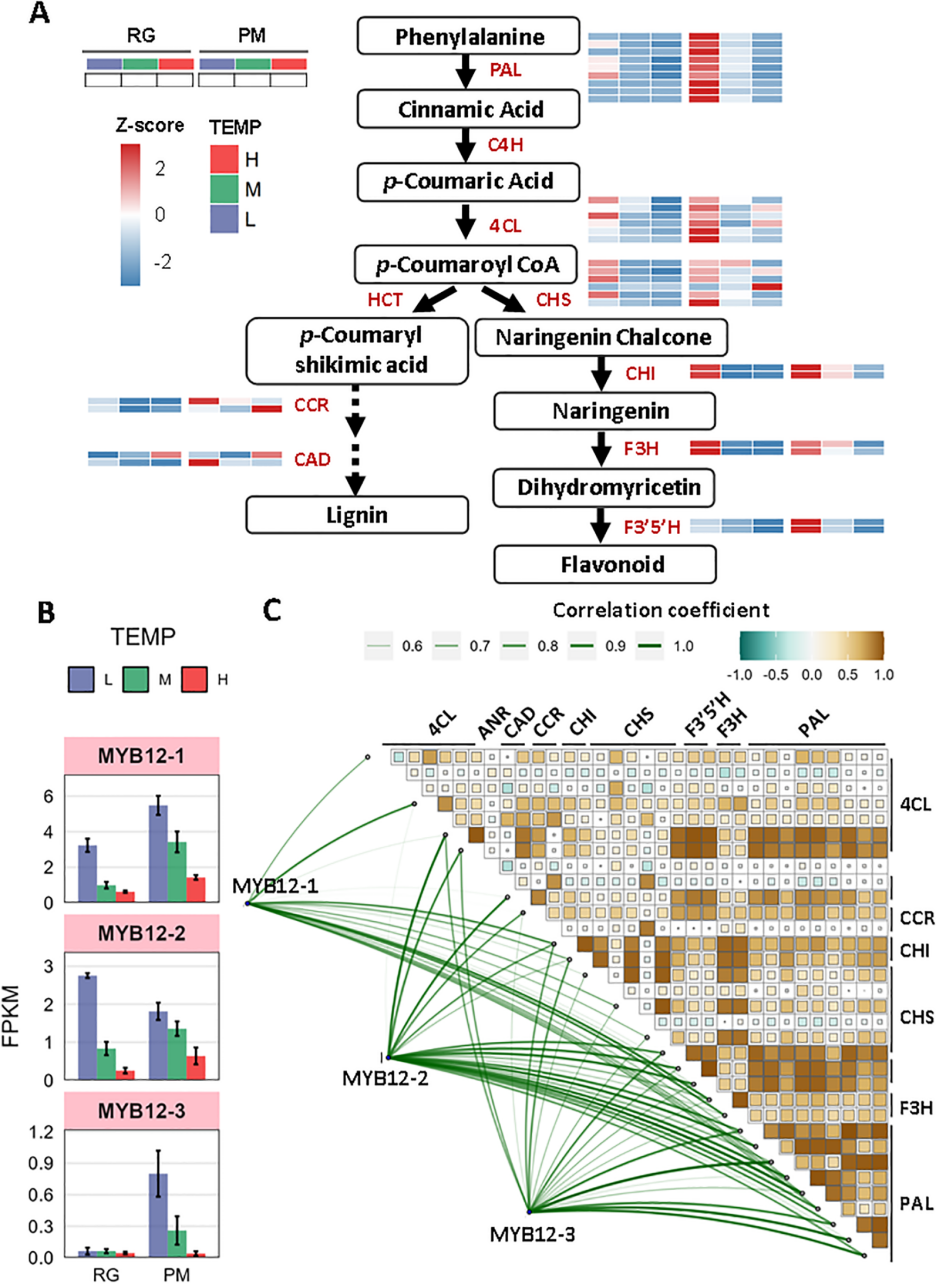
The response of phenylpropanoid biosynthesis to low and high temperature. **(A)** Diagram of phenylpropanoid metabolism pathway coupled with heatmap analysis of z-scored expression profiles of genes involved. **(B)** Transcript abundance (FPKM) of three *MYB12* TFs under low- and high- temperature. **(C)** Pearson correlation and Mantel test between the three *MYB12* TFs and DEGs involved in phenylpropanoid biosynthesis. Pearson’s correlation coefficient matrix shows the relationships among genes involved in phenylpropanoid biosynthesis. Significance levels are indicated by square size, with green indicating negative correlations and brown indicating positive ones. The line width reflects the Mantel’s r statistic for each respective correlation. The size of the line and the color of the box both represent correlation coefficients (Mantel’s r).

Phenylpropanoid biosynthesis leads to the production of a wide range of metabolites, including lignin, flavonoids, and various aromatic compounds such as coumarins, phenolic volatiles, and hydrolyzable tannins (Thomas, 2010). This study investigated the molecular regulation of polyphenolic and flavonoid biosynthesis by examining gene expression profiles within the phenylpropanoid pathway. Through heatmap visualization of row z-scored gene expression profiles, we observed that genes encoding key enzymes in this pathway-phenylalanine ammonia-lyase (*PAL*), 4-coumarate-CoA ligase (*4CL*), chalcone synthase (*CHS*), chalcone isomerase (*CHI*), and flavanone 3-hydroxylase (*F3H*) were highly expressed under low temperatures at the RG stage, compared to other conditions (**Figure 6A**). By the PM stage, this high expression pattern was more pronounced. In addition, genes for flavonoid 3’,5’-hydroxylase (*F3’5’H*), cinnamoyl-CoA reductase-1 (*CCR-1*) and cinnamyl alcohol dehydrogenase-2 (*CAD-2*) also demonstrated high expression levels. When assessing their expression relative to control conditions, we observed significant upregulation in multiple *PAL*, *CHS*, *CHI*, and *F3H* genes, with the notable exception of downregulation of *NtPAL-8* at RG stage (**Supplementary Table S7A**). However, only *NtCHS-6*, *Nt4CL-6*, *NtCHI-2*, and *NtF3’5’H-1* showed significant increases in expression levels at PM stage (**Supplementary Table S7A**). In contrast, the majority of genes involved had low expressions under high temperature. Compared to control conditions, a variety of genes encoding *PAL*, *CHS*, and *F3’5’H* demonstrated a marked reduction in expression levels after different exposure of high-temperature stress (**Supplementary Table S7A**). In conclusion, phenylpropanoid biosynthesis appeared to be positively regulated by low temperatures but negatively affected by high temperatures. To validate the transcriptome data, qRT-PCR analyses were performed on *NtPAL-5*, *NtCHS-3*, *NtCHI-2*, and *NtF3H-1*. The results confirmed that their expression patterns aligned with RNA-Seq data, demonstrating data reliability (**Supplementary Figure S2**).

Additionally, R2R3-MYBTFs, particularly *NtMYB12*, have been identified as specific activators in the phenylpropanoid biosynthesis pathway and are crucial for flavonoid synthesis (Mehrtens et al., 2005). In our study, the transcriptional levels of *NtMYB12-1*, *NtMYB12-2*, and *NtMYB12-3* were increased under low temperature but decreased under high temperature at both RG and PM stage (**Figure 6B**, **Supplementary Table S7B**). Moreover, significant positive correlations were observed between *MYB12-1/-2/-3* and genes encoding *PAL*, *4CL*, *CHS*, *CHI*, *F3H*, *F3’5’H*, *CCR*, and *CAD* (**Figure 6C**, **Supplementary Tables S7C, D**). Consequently, *NtMYB12-1*, *NtMYB12-2*, and *NtMYB12-3* are likely to function as pivotal regulatory factors for distinct enzymes in the phenylpropanoid biosynthesis pathway.

### 3.7 Identification of co-expression networks associated with phenylpropanoid biosynthesis

**Figure 7:**
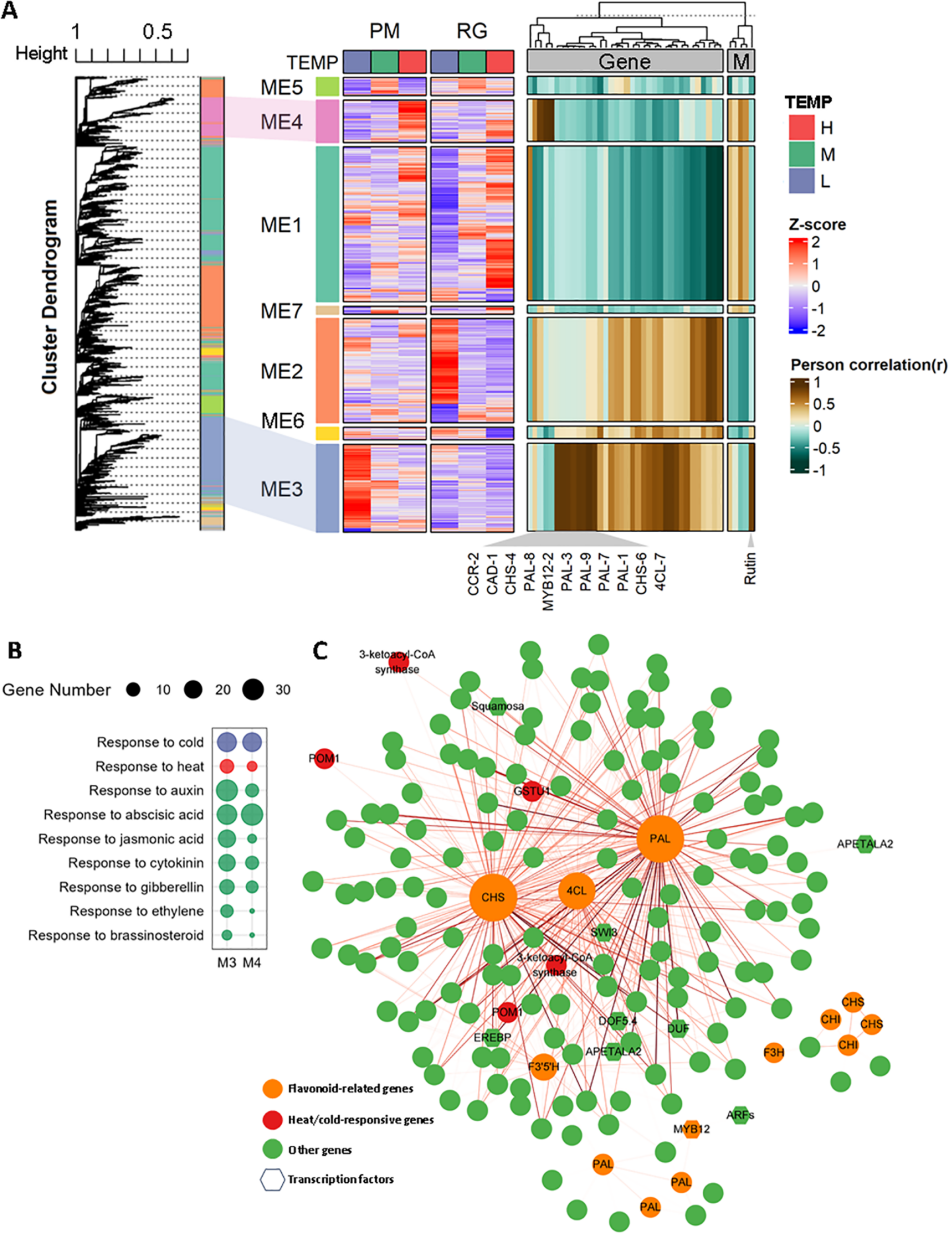
Weighted gene co-expression network analysis (WGCNA) of differentially expressed genes (DEGs) **(A)** A hierarchical cluster tree generated by WGCNA, with each leaf representing a single gene. The primary branches form seven distinct co-expression modules, each marked by a unique color. The heatmap in the middle illustrates the gene expression patterns within each module under various temperature conditions, while the heatmap on the right shows the correlations between each module and genes related to the phenylpropanoid biosynthesis pathway and polyphenolic metabolite contents. **(B)** GO enrichment analysis related to hormones for genes found in Module 3 and Module 4. **(C)** A network map depicting the co-expression relationships among genes within Modules 3 and 4.

To identify key genes involved in temperature-induced phenylpropanoid biosynthesis, a weighted correlation network analysis (WGCNA) was conducted to build a gene co-expression network. We excluded genes with low FPKM values (FPKM < 1) and utilized the dynamic tree cut method to merge genes with similar expression profiles. This analysis yielded 6,427 differentially expressed genes (DEGs) distributed across seven co-expressed gene modules (**Figure 6A**). Heatmap analysis revealed that DEGs within the same module exhibited similar expression patterns under both low and high-temperature conditions, consistent with the findings of WGCNA. Module 3 (ME3) and module 4 (ME4) showed highest positive correlations with DEGs involved in phenylpropanoid biosynthesis, therefore identified as high correlation modules (**Figure 7A**, **Supplementary Table S8**). GO terms related to hormones and responses to cold/heat stress for DEGs nested in ME3 and ME4 were examined then (**Figure 7B**). As depicted in **Figure 7B**, ME3 had a higher number of DEGs enriched in GO terms associated with “responses to auxin”, “jasmonic acid”, “cytokinin”, “ethylene”, and “brassinosteroid”, compared to ME4. The only exception was the enrichment of genes associated with the “response to ABA”, which was greater in ME4. Additionally, ME3 contained twice as many DEGs enriched in “responses to heat” compared to ME4, while the number of DEGs enriched in “responses to cold” was similar between both modules. To pinpoint hub genes associated with phenylpropanoid biosynthesis within ME3 and ME4, we performed a gene network analysis using CYTOSCAPE software. Hub genes were identified based on their eigengene connectivity (KME), reflecting the correlation between a gene’s expression value and the module’s eigengene. Genes with an absolute KME value of 0.7 or higher were classified as hub genes. Among the top 10 hub genes, *NtCHS-6*, *Nt4CL-6*, and *NtPAL-1* were selected for further study due to their relevance to phenylpropanoid biosynthesis and were supposed to play a significant role in temperature-induced phenylpropanoid biosynthesis (**Figure 7C**, **Supplementary Table S9**). The significance of these hub genes stems from their strategic enzymatic functions at critical nodes.*NtPAL-1* catalyzes the initial, committed step of the entire pathway, *NtCHS-6* mediates the first dedicated step in flavonoid biosynthesis, *Nt4CL-6* functions at a central branch point, activating substrates for various downstream pathways. Their co-expression as hubs within a single module indicates a tightly co-regulated network. This sophisticated, multi-tiered regulation ensures a balanced metabolic flux, directing resources efficiently toward the synthesis of defensive compounds such as flavonoids under temperature stress.

## 4 Discussion

### 4.1 ROS homeostasis and temperature stress

The production and scavenging of reactive oxygen species (ROS) are crucial for plant responses to abiotic stress. In response to such stress, ROS homeostasis in plant cells can be influenced by various factors, such as stress severity, duration, plant species, and tolerance levels. It has been documented that exposure to cold stress initially elevates malondialdehyde (MDA) content and the activity of antioxidant enzymes, followed by subsequent reductions (Yao et al., 2018; Quan et al., 2023). In contrast, at low temperatures (4°C), antioxidant capacity is reduced in the early stages but increases later as enhanced polyphenol and lignin biosynthesis, along with antioxidant enzymes, promoting ROS detoxification (Zhou et al., 2018). Under moderate high-temperature conditions, plants can boost their antioxidant defenses, decrease ROS accumulation, and regulate both basal and acquired thermotolerance, thereby delaying ROS-induced cell death (Wang et al., 2014; Fan et al., 2018). However, sustained or extreme high-temperature exposure may cause cellular damage in plants (Zhao et al., 2020; Hu et al., 2020). Additionally, studies have demonstrated that heat-tolerant chickpea varieties exhibit smaller increases in H_2_O_2_ and MDA levels compared to heat-sensitive ones (Awasthi et al., 2017). In our study, exposure to both low and high temperatures for different durations led to the accumulation of H_2_O_2_ and O^2−^, significantly elevating MDA levels, which aligns with findings by Yang et al. (2018). Unexpectedly, the activities of antioxidant enzymes such as SOD, POD, and CAT remained significantly suppressed, indicating that the excessive ROS generation and a weakened antioxidant system may have induced oxidative stress in the plant cells.

### 4.2 Similarities and differences in low-temperature and high-temperature responses

Previous research suggests that common adaptive mechanisms may help plants resist various types of stress. For example, extreme temperatures, whether high or low, can cause oxidative damage in plant cells, which subsequently triggers the activation of antioxidant enzyme systems (Suzuki and Mittler, 2006; Blagojevic et al., 2011). This observation suggests that certain cold-induced genes, responsible for the regulation of antioxidant enzymes or compounds, might potentially mitigate oxidative damages under heat stress conditions. Transcription factors, *CBF/DREB*, implicated in cold response, guide plants to activate common responsive process, like ROS-responsive pathway in many abiotic stresses (Yun et al., 2022). Furthermore, the phenomenon of cross-tolerance, where exposure to one type of stress enhances resistance to another, has been reported in multiple plant species (Yasuda, 2017). For instance, *OsHSP18.6* was induced under both cold and heat stress conditions in rice. Its overexpression led to enhanced heat tolerance and simultaneously resulted in improved cold tolerance (Wang et al., 2015). Additionally, under cold stress conditions, the co-expression of *HSFA2* and HSP90 activated heat stress responses, thereby enhancing plant cold stress tolerance (Yasuda, 2017). In this study, we observed that both cold and heat stress shared many upregulated and downregulated genes, including *NtRD22*, *NtPIF3*, *NtCBF1*, and *NtHSP70*, implying that these genes may represent potential targets for cross-tolerance (**Supplementary Table S10**).

Nevertheless, low and high temperatures can also trigger entirely distinct responses in plants. For instance, low temperatures cause the condensation of lipid molecules in cell membranes, rendering the membrane more solid and viscous, while high temperatures increased lipid fluidity, making the membrane more fluid (Sangwan et al., 2002). To identify robust molecules that might perceived high or low temperature signals and react differently in plants, we examine DEGs with opposite expression patterns under high-temperature and low-temperature stress (**Supplementary Table S10**). The transcription factor *ABF2* was significantly downregulated under cold stress but markedly upregulated under heat stress, which was consistent with findings that *ABF2* was downregulated in cold-tolerant rice varieties (Shen et al., 2022) and overexpression of *ABF2* in plants conferred heat tolerance (Kim et al., 2004). Furthermore, this study observed upregulation of the calcium ion sensor molecule CIPK (Calmodulin binding protein-like) under heat stress and downregulated under cold stress. Previous research has implicated CIPK in sodium ion efflux in plants, thereby conferring salt tolerance (Amarasinghe et al., 2016), suggesting its potential crucial role in clod and heat responses. These datasets might help us identify sensors that enable plants to perceive temperature changes, prompting distinct responses to either low or high temperatures.

### 4.3 Polyphenolic biosynthesis and cold/heat response

Polyphenolic components in tobacco primarily encompass tannins (such as chlorogenic acid and caffeic acid), coumarins (e.g., scopoletin), and flavonoids (quercetin and anthocyanins) (Zou et al., 2021). These compounds can mitigate membrane lipid peroxidation and reduce cellular damage under cold stress. Flavonoids are hypothesized to confer protection to plants under cold stress, potentially by scavenging ROS through their inherent antioxidant capabilities (Schulz et al., 2016). And antioxidant flavonoids significantly enhanced oxidative and drought tolerance in *Arabidopsis* (Nakabayashi et al., 2014). Furthermore, flavonoids are considered essential for protecting cell membranes and proteins from damage during freezing injuries, highlighting their dual role as both ROS scavengers and cellular protectors (Schulz et al., 2016). In this study, prolonged exposure to mild low temperatures significantly enhanced flavonoid accumulation in CTLs, likely due to the upregulation of the phenylpropanoid biosynthesis pathway and *NtMYB12-1/-2/-3*. This enhanced flavonoid biosynthesis was hypothesized to serve as an adaptive mechanism under low temperatures. Moreover, low temperature exposure led to an increase in total phenolic content, aligning with observations made by Gu et al. (2021). In contrast, unchanged or decreased polyphenol levels and subsequently transient or sustained increases in lignin biosynthesis were found upon exposure to low temperatures at 4°C in tobacco leaves, due to polyphenolic compound degradation towards lignin synthesis, thereby reinforcing the cell wall and bolstering plant resistance (Yang et al., 2015; Zhou et al., 2018; Šamec et al., 2021). These observed discrepancies may be ascribed to the application of lower temperature conditions in these experiments, potentially leading to more significant plant damages, or may reflect the short-term nature of the applied stress.

Previous studies have reported that plants produce increased levels of phenolic compounds, such as anthocyanins, flavonoids, and phenolic acids, as a defensive response to high temperatures, in order to protect their cells from damage (Ancillotti et al., 2015; Zlotek et al., 2015; Martinez et al., 2016). Enhanced accumulation of these phenolic compounds has been linked to improved heat tolerance in *F. trachyphylla* plants (Wang et al., 2019). In carrots, the buildup of phenolics, including anthocyanins, is thought to safeguard against oxidative damage triggered by heat stress (Commisso et al., 2016). In contrast, our investigation revealed that flavonoid levels remained constant, whereas chlorogenic acid content experienced a significant reduction. Unlike the substantial upregulation of key genes associated with phenylpropanoid biosynthesis under low-temperature conditions, only a limited number of genes were downregulated in response to high temperatures. This led us to hypothesize that the regulation of the polyphenolic biosynthetic pathway under conditions of high-temperature stress might be predominantly influenced by activity levels rather than transcriptional changes.

## 5 Conclusion

In summary, our study demonstrates that long-term mild temperature stress induces significant oxidative damage in cigar tobacco leaves, as evidenced by ROS accumulation and a lack of induction in major antioxidant enzyme activities. Counterintuitively, under low-temperature conditions, we observed a substantial accumulation of phenolic and flavonoid compounds. Our multi-omics analysis reveals that this response is governed by a profound metabolic reprogramming centered on the phenylpropanoid biosynthesis pathway. This pathway exhibits a divergent regulatory pattern, being potently activated under cold stress but suppressed under heat. Mechanistically, this differential response is orchestrated by the NtMYB12 transcription factors and is mediated by the coordinated expression of key structural genes, particularly the hub genes *NtPAL-1*, *NtCHS-6*, and *Nt4CL-6* identified via co-expression network analysis. These hub genes, functioning as master regulators at critical metabolic nodes, facilitate the efficient channeling of carbon flux toward the synthesis of protective polyphenols, thereby mitigating oxidative damage and ultimately determining leaf quality under temperature stress. Taken together, this research provides insights on understanding the response of cigar plants to temperature stress and reference for selecting high-quality cigar planting area (e.g. appropriate environmental temperature) in China.

## Data Availability

The datasets presented in this study can be found in online repositories. The names of the repository/repositories and accession number(s) can be found below: https://www.ncbi.nlm.nih.gov/, PRJNA955170.
