# Supplementary material for: Study on mechanism of temperature-modulated polyphenolic biosynthesis in cigar tobacco leaves
Source: Front Plant Sci. 2025 Oct 23;16:1693512. doi: 10.3389/fpls.2025.1693512 (PMC12589083; doi:10.3389/fpls.2025.1693512)
Supplement: Supplementary file 1 [file DataSheet1.zip › supplementary materials/Supplementary figures.pdf]

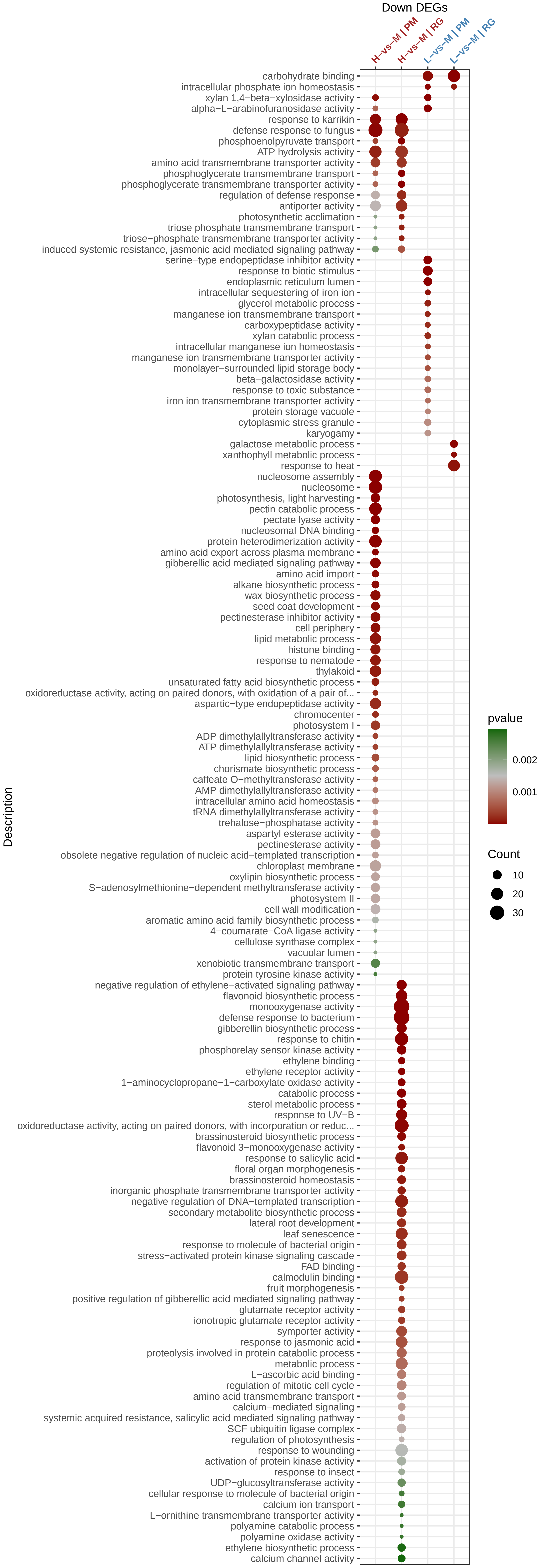
Down DEGs
H−vs−M | PM
H−vs−M | RG
L−vs−M | PM
L−vs−M | RG
Description
carbohydrate binding
intracellular phosphate ion homeostasis
xylan 1,4−beta−xylosidase activity
alpha−L−arabinofuranosidase activity
response to karrikin
defense response to fungus
phosphoenolpyruvate transport
ATP hydrolysis activity
amino acid transmembrane transporter activity
phosphoglycerate transmembrane transport
phosphoglycerate transmembrane transporter activity
regulation of defense response
antiporter activity
photosynthetic acclimation
triose phosphate transmembrane transport
triose−phosphate transmembrane transporter activity
induced systemic resistance, jasmonic acid mediated signaling pathway
serine−type endopeptidase inhibitor activity
response to biotic stimulus
endoplasmic reticulum lumen
intracellular sequestering of iron ion
glycerol metabolic process
manganese ion transmembrane transport
carboxypeptidase activity
xylan catabolic process
intracellular manganese ion homeostasis
manganese ion transmembrane transporter activity
monolayer−surrounded lipid storage body
beta−galactosidase activity
response to toxic substance
iron ion transmembrane transporter activity
protein storage vacuole
cytoplasmic stress granule
karyogamy
galactose metabolic process
xanthophyll metabolic process
response to heat
nucleosome assembly
nucleosome
photosynthesis, light harvesting
pectin catabolic process
pectate lyase activity
nucleosomal DNA binding
protein heterodimerization activity
amino acid export across plasma membrane
gibberellic acid mediated signaling pathway
amino acid import
alkane biosynthetic process
wax biosynthetic process
seed coat development
pectinesterase inhibitor activity
cell periphery
lipid metabolic process
histone binding
response to nematode
thylakoid
unsaturated fatty acid biosynthetic process
oxidoreductase activity, acting on paired donors, with oxidation of a pair of...
aspartic−type endopeptidase activity
chromocenter
photosystem I
ADP dimethylallyltransferase activity
ATP dimethylallyltransferase activity
lipid biosynthetic process
chorismate biosynthetic process
caffeate O−methyltransferase activity
AMP dimethylallyltransferase activity
intracellular amino acid homeostasis
tRNA dimethylallyltransferase activity
trehalose−phosphatase activity
aspartyl esterase activity
pectinesterase activity
obsolete negative regulation of nucleic acid−templated transcription
chloroplast membrane
oxylipin biosynthetic process
S−adenosylmethionine−dependent methyltransferase activity
photosystem II
cell wall modification
aromatic amino acid family biosynthetic process
4−coumarate−CoA ligase activity
cellulose synthase complex
vacuolar lumen
xenobiotic transmembrane transport
protein tyrosine kinase activity
negative regulation of ethylene−activated signaling pathway
flavonoid biosynthetic process
monooxygenase activity
defense response to bacterium
gibberellin biosynthetic process
response to chitin
phosphorelay sensor kinase activity
ethylene binding
ethylene receptor activity
1−aminocyclopropane−1−carboxylate oxidase activity
catabolic process
sterol metabolic process
response to UV−B
oxidoreductase activity, acting on paired donors, with incorporation or reduc...
brassinosteroid biosynthetic process
flavonoid 3−monooxygenase activity
response to salicylic acid
floral organ morphogenesis
brassinosteroid homeostasis
inorganic phosphate transmembrane transporter activity
negative regulation of DNA−templated transcription
secondary metabolite biosynthetic process
lateral root development
leaf senescence
response to molecule of bacterial origin
stress−activated protein kinase signaling cascade
FAD binding
calmodulin binding
fruit morphogenesis
positive regulation of gibberellic acid mediated signaling pathway
glutamate receptor activity
ionotropic glutamate receptor activity
symporter activity
response to jasmonic acid
proteolysis involved in protein catabolic process
metabolic process
L−ascorbic acid binding
regulation of mitotic cell cycle
amino acid transmembrane transport
calcium−mediated signaling
systemic acquired resistance, salicylic acid mediated signaling pathway
SCF ubiquitin ligase complex
regulation of photosynthesis
response to wounding
activation of protein kinase activity
response to insect
UDP−glucosyltransferase activity
cellular response to molecule of bacterial origin
calcium ion transport
L−ornithine transmembrane transporter activity
polyamine catabolic process
polyamine oxidase activity
ethylene biosynthetic process
calcium channel activity
pvalue
0.002
0.001
Count
10
20
30

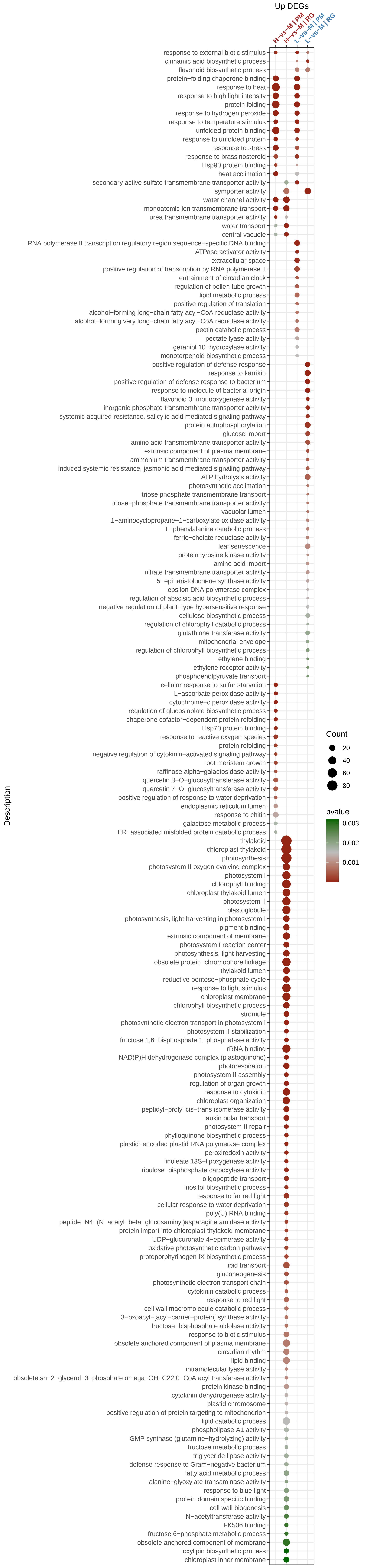
Up DEGs
H−vs−M | PM
H−vs−M | RG
L−vs−M | PM
L−vs−M | RG
Description
response to external biotic stimulus
cinnamic acid biosynthetic process
flavonoid biosynthetic process
protein−folding chaperone binding
response to heat
response to high light intensity
protein folding
response to hydrogen peroxide
response to temperature stimulus
unfolded protein binding
response to unfolded protein
response to stress
response to brassinosteroid
Hsp90 protein binding
heat acclimation
secondary active sulfate transmembrane transporter activity
symporter activity
water channel activity
monoatomic ion transmembrane transport
urea transmembrane transporter activity
water transport
central vacuole
RNA polymerase II transcription regulatory region sequence−specific DNA binding
ATPase activator activity
extracellular space
positive regulation of transcription by RNA polymerase II
entrainment of circadian clock
regulation of pollen tube growth
lipid metabolic process
positive regulation of translation
alcohol−forming long−chain fatty acyl−CoA reductase activity
alcohol−forming very long−chain fatty acyl−CoA reductase activity
pectin catabolic process
pectate lyase activity
geraniol 10−hydroxylase activity
monoterpenoid biosynthetic process
positive regulation of defense response
response to karrikin
positive regulation of defense response to bacterium
response to molecule of bacterial origin
flavonoid 3−monooxygenase activity
inorganic phosphate transmembrane transporter activity
systemic acquired resistance, salicylic acid mediated signaling pathway
protein autophosphorylation
glucose import
amino acid transmembrane transporter activity
extrinsic component of plasma membrane
ammonium transmembrane transporter activity
induced systemic resistance, jasmonic acid mediated signaling pathway
ATP hydrolysis activity
photosynthetic acclimation
triose phosphate transmembrane transport
triose−phosphate transmembrane transporter activity
vacuolar lumen
1−aminocyclopropane−1−carboxylate oxidase activity
L−phenylalanine catabolic process
ferric−chelate reductase activity
leaf senescence
protein tyrosine kinase activity
amino acid import
nitrate transmembrane transporter activity
5−epi−aristolochene synthase activity
epsilon DNA polymerase complex
regulation of abscisic acid biosynthetic process
negative regulation of plant−type hypersensitive response
cellulose biosynthetic process
regulation of chlorophyll catabolic process
glutathione transferase activity
mitochondrial envelope
regulation of chlorophyll biosynthetic process
ethylene binding
ethylene receptor activity
phosphoenolpyruvate transport
cellular response to sulfur starvation
L−ascorbate peroxidase activity
cytochrome−c peroxidase activity
regulation of glucosinolate biosynthetic process
chaperone cofactor−dependent protein refolding
Hsp70 protein binding
response to reactive oxygen species
protein refolding
negative regulation of cytokinin−activated signaling pathway
root meristem growth
raffinose alpha−galactosidase activity
quercetin 3−O−glucosyltransferase activity
quercetin 7−O−glucosyltransferase activity
positive regulation of response to water deprivation
endoplasmic reticulum lumen
response to chitin
galactose metabolic process
ER−associated misfolded protein catabolic process
thylakoid
chloroplast thylakoid
photosynthesis
photosystem II oxygen evolving complex
photosystem I
chlorophyll binding
chloroplast thylakoid lumen
photosystem II
plastoglobule
photosynthesis, light harvesting in photosystem I
pigment binding
extrinsic component of membrane
photosystem I reaction center
photosynthesis, light harvesting
obsolete protein−chromophore linkage
thylakoid lumen
reductive pentose−phosphate cycle
response to light stimulus
chloroplast membrane
chlorophyll biosynthetic process
stromule
photosynthetic electron transport in photosystem I
photosystem II stabilization
fructose 1,6−bisphosphate 1−phosphatase activity
rRNA binding
NAD(P)H dehydrogenase complex (plastoquinone)
photorespiration
photosystem II assembly
regulation of organ growth
response to cytokinin
chloroplast organization
peptidyl−prolyl cis−trans isomerase activity
auxin polar transport
photosystem II repair
phylloquinone biosynthetic process
plastid−encoded plastid RNA polymerase complex
peroxiredoxin activity
linoleate 13S−lipoxygenase activity
ribulose−bisphosphate carboxylase activity
oligopeptide transport
inositol biosynthetic process
response to far red light
cellular response to water deprivation
poly(U) RNA binding
peptide−N4−(N−acetyl−beta−glucosaminyl)asparagine amidase activity
protein import into chloroplast thylakoid membrane
UDP−glucuronate 4−epimerase activity
oxidative photosynthetic carbon pathway
protoporphyrinogen IX biosynthetic process
lipid transport
gluconeogenesis
photosynthetic electron transport chain
cytokinin catabolic process
response to red light
cell wall macromolecule catabolic process
3−oxoacyl−[acyl−carrier−protein] synthase activity
fructose−bisphosphate aldolase activity
response to biotic stimulus
obsolete anchored component of plasma membrane
circadian rhythm
lipid binding
intramolecular lyase activity
obsolete sn−2−glycerol−3−phosphate omega−OH−C22:0−CoA acyl transferase activity
protein kinase binding
cytokinin dehydrogenase activity
plastid chromosome
positive regulation of protein targeting to mitochondrion
lipid catabolic process
phospholipase A1 activity
GMP synthase (glutamine−hydrolyzing) activity
fructose metabolic process
triglyceride lipase activity
defense response to Gram−negative bacterium
fatty acid metabolic process
alanine−glyoxylate transaminase activity
response to blue light
protein domain specific binding
cell wall biogenesis
N−acetyltransferase activity
FK506 binding
fructose 6−phosphate metabolic process
obsolete anchored component of membrane
oxylipin biosynthetic process
chloroplast inner membrane
Count
20
40
60
80
pvalue
0.003
0.002
0.001

Figure.S1 Enrichment analysis of GO terms response to high temperature (H) and low temperature (L) stress.

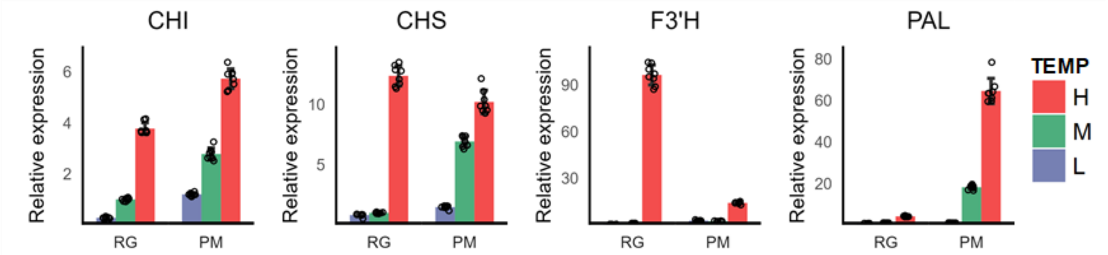

Figure. S2 qPCR analysis of key gene in phenylpropanoid biosynthesis.

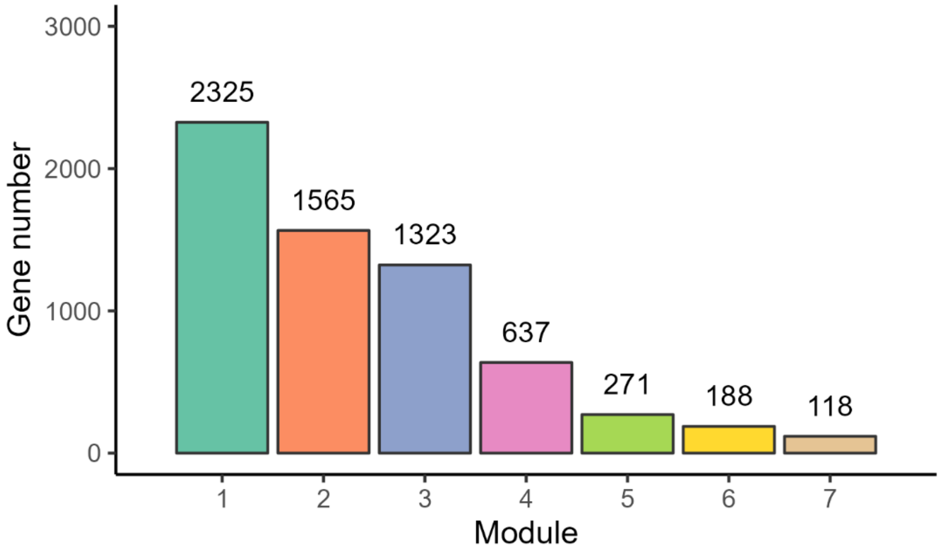

Figure. S3(A) Gene numbers clustered in different modules.

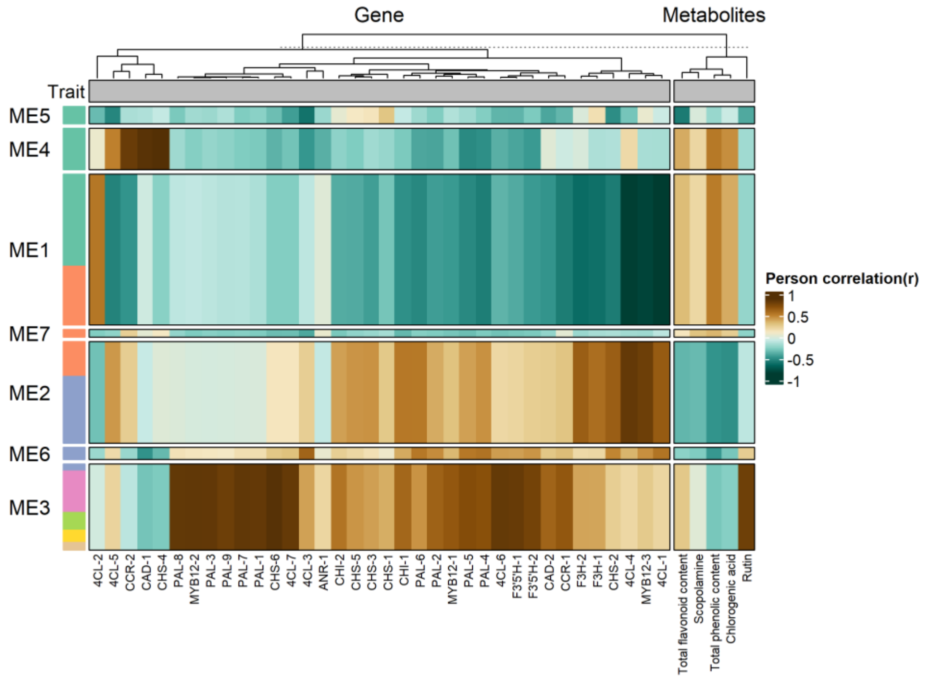

Figure. S3(B) The correlation heatmap show the Pearson coefficient for genes and their modules clustered.
